# Efficiency and hemodynamics of dual drainage venovenous ECMO: A computational parametric study

**DOI:** 10.1371/journal.pone.0354915

**Published:** 2026-08-04

**Authors:** Monica Emendi, Hanna Hörwing, Louis Parker, Anders Svensson Marcial, Torkel Brismar, Lars Mikael Broman, Lisa Prahl Wittberg

**Affiliations:** 1 Department of Engineering Mechanics, KTH Royal Institute of Technology, Stockholm, Sweden; 2 School of Engineering, University of Western Australia, Perth, Australia; 3 Department of Radiology, Karolinska University Hospital and Karolinska Institutet, Stockholm, Sweden; 4 Department of Clinical Science, Intervention and Technology, Karolinska Institutet, Stockholm, Sweden; 5 ECMO Centre Karolinska, Pediatric Perioperative Medicine and Intensive Care, Karolinska University Hospital, Stockholm, Sweden; 6 Department of Physiology and Pharmacology, Karolinska Institutet, Stockholm, Sweden; Scuola Superiore Sant’Anna, ITALY

## Abstract

Venovenous extracorporeal membrane oxygenation (VV ECMO) can be performed with different cannulation approaches: femoro-femoral (FF), femoro-jugular (FJ), or jugulo-femoral (JF), all characterized by one drainage cannula. Dual drainage cannulation (JFF, with two drainage cannulae) may be considered in refractory hypoxemia. This work compared single vs. dual drainage concerning the impact on hemodynamics and oxygenation performance by studying the drainage ratio between the jugular and femoral cannulae, their type and position. Computational fluid dynamics was used in a patient-averaged model of the right atrium (RA) and central veins to estimate recirculation fraction (R${\;}_{f}$), arterial oxygen saturation (SaO${\;}_{2}$), caval pressures, shear rates, time-averaged wall shear stress (TAWSS), and stagnation volume in each cannulation configuration. An ECMO flow rate of 4 L/min and cardiac output of 6 L/min were considered. JF showed the highest R${\;}_{f}$ (22%) and the lowest SaO${\;}_{2}$ (80%). The lowest R${\;}_{f}$ (0.03%) and the highest SaO${\;}_{2}$ (90%) were obtained in JFF with a multistage jugular cannula draining at a flow rate lower than the native venous inflow. FF presented the lowest pressure in the inferior vena cava (IVC, −10 mmHg), but the highest in the superior vena cava (SVC, 32 mmHg) when the return cannula tip was placed at the superior cavo-atrial junction. The stagnation volume was highest in JFF at femoral drainage ≤2 L/min. In most configurations the maximum TAWSS in the RA and SVC were more than 5 times higher than no ECMO (baseline). Adding an additional drainage cannula may in some cases improve oxygenation compared to standard single drainage configurations. The efficiency of JFF was mainly influenced by the jugular/femoral drainage ratio, cannula type and position in the SVC. Significant differences in caval pressures and TAWSS were observed between the configurations. The optimal choice of cannulation would be patient-tailored, considering specific needs and complication risks.

## Introduction

Extracorporeal membrane oxygenation (ECMO) is a lifesaving treatment for the critically ill when conventional intensive care does not suffice. When respiratory support is needed, venovenous (VV) ECMO may be employed. One critical clinical parameter for high efficiency in VV ECMO is a low recirculation fraction (R${\;}_{f}$), defined as the amount of returned oxygenated blood directly withdrawn by the drainage cannula, thus not contributing to the patient oxygenation [1]. Gagliardi et al. reported a R${\;}_{f}$ of 10–30% to be clinically acceptable [2]. However, there is no established threshold for high recirculation, while the Extracorporeal Life Support Organization (ELSO, Ann Arbor, Mi, US) guidelines recommend an arterial oxygen saturation (SaO${\;}_{2}$) above 80% [3]. The main complications related to VV ECMO include bleeding, thrombosis [4–6] and acute renal failure [7,8].

There are different basic configurations in multi-site cannulation using single-lumen cannulae for VV ECMO that depend on the site of insertion of the drainage and return cannulae: femoro-femoral (FF), femoro-jugular (FJ) and jugulo-femoral (JF) [9]. These are referred to as single drainage cannulation. In some cases, a single drainage configuration is not enough, e.g., in refractory hypoxemia. In this situation, a few clinical studies suggested to add a second drainage cannula to improve oxygenation, although increasing the risk of infection and vessel injury [10–12]. This cannulation strategy, referred to as dual drainage, bicaval drainage or VV-V ECMO, can be obtained from modifications to the ECMO circuit by introducing a Y-piece connector to connect the additional drainage cannula tubing. The addition of a drainage cannula, from either JF or FF configuration, has improved the oxygenation support in several clinical cases [10,11]. Charbit et al. proposed a mathematical model to simulate the dual drainage cannulation and found higher levels of systemic oxygenation for a high ratio of ECMO flow rate/cardiac output and in the presence of a pulmonary shunt [12]. Several retrospective clinical studies investigating the impact of cannulation on oxygenation performance and ECMO outcomes for single drainage configurations called for further randomized trials [13–16].

The hemodynamics of single drainage VV or veno-arterial (VA) ECMO configurations has been studied in previous works by means of computational fluid dynamics (CFD) [17–19]. Parker et al. found good agreement with clinical data in terms of R${\;}_{f}$ for FJ and JF configurations, concluding that R${\;}_{f}$ was mostly influenced by the availability of native venous blood into the drainage zone [17]. Another parameter that could possibly influence R${\;}_{f}$ is the position of the cannulae. According to CFD studies [17] and clinical data [20], moving the drainage cannula in JF up in the superior vena cava (SVC) had no significant impact on R${\;}_{f}$. Regarding FJ, a retrospective clinical study on 278 patients suggested that placing the drainage cannula in the right atrium (RA) instead of in the inferior vena cava (IVC) was more effective in terms of fluid management. Oxygenation, on the other hand, was not significantly affected [21]. The latter is in line with CFD findings from Leoni et al., concluding that the position of the drainage cannula did not significantly affect oxygen delivery [19]. Moreover, recent computational studies have shown that the flow features developing due to ECMO are characterized by high shear stress and regions of stasis, which may contribute to blood damage, platelet activation and thrombus formation [22,23], the latter supported by clinical observations of cannula-related thrombosis [24,25]. Other possible issues related to the altered fluid dynamics of VV ECMO configurations include changes of the IVC pressure, where low negative values may lead to vein collapse and cannula chattering [26,27]. Cannula obstruction of the vascular lumen along with low drainage flow rate and/or concomitant return flow rate (alongside the venous native flow) may lead to an increase in venous pressure. This in turn may result in edema formation (if pressure increases in SVC), or decreased organ perfusion pressure (if pressure increases in IVC), for example increasing the risk of acute kydney injury in case the renal venous pressure increases [28,29].

Comprehensive studies regarding dual drainage cannulation are lacking, both from clinical and fluid mechanical perspective. The influence of drainage ratio, cannulae type and position on oxygenation performance and hemodynamic parameters is yet to be investigated. Therefore, the present study aimed to elucidate the conditions where adding a second drainage cannula could be beneficial to oxygenation, using CFD to assess the related hemodynamic changes, and comparing with single drainage configurations. To increase knowledge in ECMO cannulation and support informed clinical decision making, we focused on the following hypothesis: a) different cannulation configurations (dual vs. single drainage) lead to different flow conditions, stress patterns and pressure values, thus influencing the complication risks, and b) the oxygenation efficiency is affected by the cannulation configuration, cannula design and position, and drainage ratio (in dual drainage cases).

## Materials and methods

### Patient-averaged geometry and cannulation configurations

The anatomy of interest (comprising the RA, the SVC, the IVC, the renal and hepatic veins and the coronary sinus) was obtained from a patient-averaged CT angiography model based on four healthy subjects, detailed in previous works [30,31](images were acquired between August and December 2019). The study was approved by Swedish Ethical Review Authority (Ethical permit 2018/438–31). The subjects gave written informed consent to participate in the study. 3D geometries of commercially available cannulae were reconstructed along the centerline of the vessels. The geometrical details (cross-sectional areas of the tricuspid valve and venous inlets, and depth of insertion of the cannulae) are given in the supplementary material (S1 File).

The ECMO flow rate, e.g., cannula flow, was set to 4 L/min and the cardiac output (CO) to 6 L/min, motivated by being common average values in VV ECMO.

For clarity, we refer to FF, FJ, and JF as single drainage configurations, whereas all dual drainage configurations are collectively referred to as JFF configurations.

#### Cannulae type.

The type of cannulae and positioning were suggested according to clinical praxis. The following configurations were modeled:

- **single drainage cannulation: femoro-femoral (FF), jugulo-femoral (JF), femoro-jugular (FJ)**. In FF and FJ, a 25Fr/55 cm multistage Maquet HLS (Getinge, Rastatt, Germany) drainage cannula was positioned in the IVC via the left femoral vein (LFV). In JF configuration, a 25Fr/38 cm multistage Maquet HLS drainage cannula was inserted in the SVC via the right internal jugular vein. For the return flow, a 19Fr/18 cm Bio-Medicus Flex (Medtronic, Tolochenaz, Switzerland) cannula was used for both the JF, and FJ configurations. In FF, a 21Fr/55 cm Bio-Medicus Flex XL (Medtronic) was used for return.
- **Dual drainage (JFF)** in which an additional drainage cannula was added to the FF configuration. Two different cases were modeled based on the type of **jugular cannula**: JFF**ss** with a 19Fr/18 cm Bio-Medicus Flex **single stage** cannula, and JFF**ms** with a 23Fr/38 cm Maquet HLS **multistage** cannula. For femoral drainage, the 25Fr/55 cm Maquet HLS cannula used in FF was replaced with the 23Fr/55 cm cannula.

The details of the drainage and return cannulae used in each scenario are summarized in Table 1. A baseline case without cannulae was also simulated for comparative analyses.

**Table 1:** Type and size of drainage and return cannulae for each configuration.

Configurations	Drainage cannula		Return cannula
	Jugular	Femoral	
FF	–	ms 25Fr/55 cm (6)	ss 21Fr/55 cm (3)
JF	ms 25Fr/38 cm (5)	–	ss 19Fr/18 cm (3)
FJ	–	ms 25Fr/55 cm (6)	ss 19Fr/18 cm (3)
JFF**ss**	**ss** 19Fr/18 cm (3)	ms 23Fr/55 cm (5)	ss 21Fr/55 cm (3)
JFF**ms**	**ms** 23Fr/38 cm (4)	ms 23Fr/55 cm (5)	ss 21Fr/55 cm (3)

*Abbreviations*: **ms**, multistage HLS cannula (Getinge, Rastatt, Germany); **ss**, single stage Bio-Medicus cannula (Medtronic, Tolochenaz, Switzerland). The sizes of the cannulae are indicated by their outer diameter in Fr (1Fr = 1/3 mm) and maximum insertion length (cm). The number of rows of side holes is reported in brackets.

#### Drainage ratio (for dual drainage configurations).

**Fig 1:**
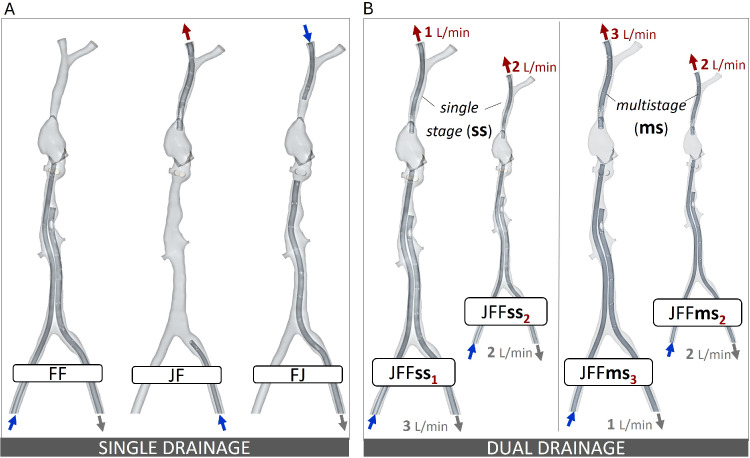
Overview of the cannulation configurations. A) Single drainage cases: femoro-femoral (FF), jugulo-femoral (JF) and femoro-jugular (FJ). B) Dual drainage cases (JFF) with single stage (JFF**ss**) and multistage (JFF**ms**) jugular cannula. Red arrows and red subscripts in the abbreviations indicate the flow rates drained by the jugular cannula, grey arrows indicate the flow rates drained by the femoral cannula, blue arrows indicate the return ECMO flow rates.

Given the hydraulic resistances of the considered cannulae and the uncertainties related to the resistance of the ECMO circuit (e.g., due to the connections between the cannulae and the circuit), different drainage ratios between the jugular and femoral cannulae were investigated in JFF cases:


$$\begin{array}{c} Drainage\;ratio=\frac{Q_{drainage\;\text{jugular}}}{Q_{drainage\;\text{femoral}}}, \end{array}$$ (1)

where $Q_{drainage\;\text{jugular}}$ and $Q_{drainage\;\text{femoral}}$ indicate respectively the jugular and femoral drainage flow rate in L/min. In detail, the single stage jugular cannula drained either 1 L/min in JFF**ss**${\;}_{1}$(dominant femoral drainage), or 2 L/min in JFF**ss**${\;}_{2}$ (equal drainage); while the multistage jugular cannula drained either 2 L/min in JFF**ms**${\;}_{2}$ (equal drainage), or 3 L/min in JFF**ms**${\;}_{3}$ (dominant jugular drainage), as described in Fig 1B. For conciseness, the subscripts are related to the jugular cannula only (being next to the type specification of jugular cannula). The relative information have been summarized in Table 2.

**Table 2:** Drainage ratio in dual drainage (JFF) configurations.

Configurations	Drainage flow rate [L/min]	
	Jugular cannula	Femoral cannula
JFF**ss**${\;}_{\text{1}}$	**1** (single stage)	3
JFF**ss**${\;}_{\text{2}}$	**2** (single stage)	2
JFF**ms**${\;}_{\text{2}}$	**2** (multistage)	2
JFF**ms**${\;}_{\text{3}}$	**3** (multistage)	1

Moreover, all the configurations described above are summarized in Fig 1.

#### Cannulae repositioning.

**Fig 2:**
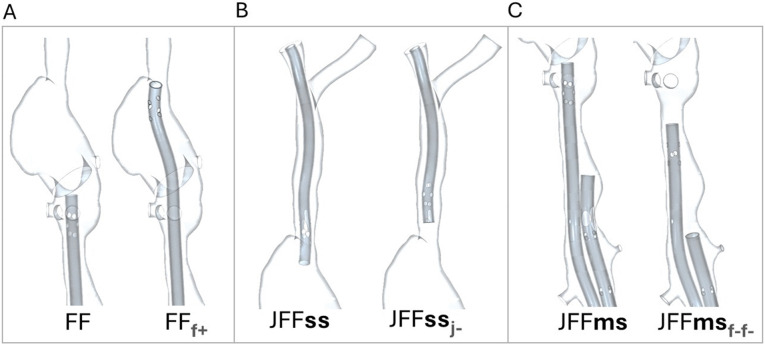
Cannulae repositioning. A) From femoro-femoral (FF), the return cannula was pushed 7 cm towards the superior cavo-atrial junction (FF${\;}_{f+}$). B) Dual drainage cases with single stage jugular cannula retracted 2 cm (JFF**ss**${\;}_{j-}$). C) From dual drainage configurations with multistage jugular cannula, the femoral and the return cannulae were retracted 3.5 cm (JFF**ms**${\;}_{f-f-}$).

To explore the impact of cannulae repositioning, the insertion depths of the cannulae were modified as follows:

- from FF, the return cannula was pushed 7 cm towards the superior cavo-atrial junction (FF${\;}_{f+}$), Fig 2A;
- from JFF**ss**, the single stage jugular cannula was retracted 2 cm upwards in the SVC (JFF**ss**${\;}_{j-}$), Fig 2B;
- from JFF**ms**, the return and the femoral drainage cannulae were retracted (pulled down) 3.5 centimeters in the IVC (JFF**ms**${\;}_{f-f-}$), Fig 2C.

### Computational methodology

Large eddy simulations (LES) were performed for each configuration using the commercial solver STAR-CCM+ (Version 2210, Siemens, Munich, Germany). A non-Newtonian Quemada viscosity model [32] with a density of 1050 kg/*m*^3^ and a hematocrit of 35% was considered to model blood. The fluid domain was discretized with polyhedral cells with prismatic boundary layer refinement. A grid convergence study was carried out considering three different mesh sizes (S1 File). The medium size was chosen as a trade-off between accuracy and computational time. Further details on the CFD setup are described in S1 File.

#### Boundary conditions.

The cardiac output was distributed as a steady flow rate between the different venous inlets (hepatic, renal, iliac, brachial) and coronary sinus according to previous works [31]. In detail, the venous return was imposed to come via the coronary sinus (2%), SVC (38%) and IVC (60%), and subsequently split among the venous branches according to their inlet cross-sectional area, as indicated in Table 3. A zero pressure outlet was set at the tricuspid valve.

**Table 3:** Boundary conditions at the inlets of the model.

	Venous inflow [% of CO]	Venous inflow [L/min]
Right brachiocephalic vein	20	1.20
Left brachiocephalic vein	18	1.08
Hepatic veins (3 inlets)	25	1.50
Coronary sinus	2	0.12
Right renal vein	16	0.96
Left renal vein	4	0.24
Right femoral vein	9	0.54
Left femoral vein	6	0.36

#### Oxygenation and hemodynamic parameters.

The recirculation fraction was calculated by introducing a passive scalar, ϕ, equal to one at the return inlet and zero elsewhere within the computational domain [31]:


$$\begin{array}{c} R_{f}=100\phi , \end{array}$$ (2)

with ϕ calculated at the drainage outlets.

To estimate the level of oxygen saturation, the following equation was used, assuming a venous blood saturation of 70% [19]:


$$\begin{array}{c} SaO_{2}=70+30\phi , \end{array}$$ (3)

calculating ϕ at the level of the tricuspid valve.

The surface-averaged, maximum and minimum values of pressure developing at the SVC and IVC walls in the different configurations were compared.

The scalar shear rate, $\dot{\gamma }$ was calculated as follows [33]:


$$\begin{array}{c} \dot{\gamma }=\sqrt{2𝐃:𝐃}, \end{array}$$ (4)

where: indicates the double dot product and **D** is the strain rate tensor, defined as:


$$\begin{array}{c} 𝐃=1/2(\nabla 𝐮+\nabla {𝐮}^{T}), \end{array}$$ (5)

being ∇𝐮 the velocity gradient. Moreover, the magnitude of wall shear stresses averaged over the last second of simulation, referred to as TAWSS, was compared for each cannulation configuration.

According to previous works, the regions with average velocity ***u*** < 1 mm/s and average strain rate $\dot{\gamma }<$2 *s*^-1^ were considered as stagnant flow zones, potentially linked to higher risk of thrombus development [22,34].

All the above-mentioned parameters were calculated averaging the values over the last second of the simulations (which ran for 3 s of physical time, to reach convergence).

## Results

### Recirculation fraction and oxygen saturation

**Fig 3:**
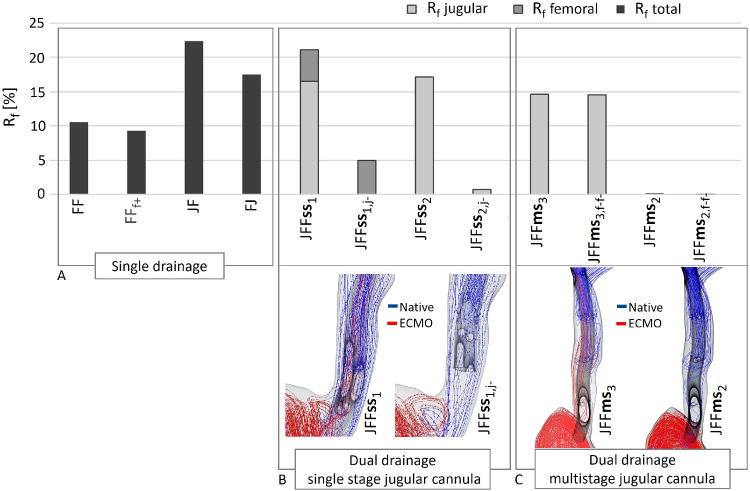
Recirculation fraction, R${\;}_{𝐟}$. Values of R${\;}_{f}$ for single drainage configurations (A), dual drainage with single stage jugular cannula (B), and with multistage jugular cannula (C). Streamlines of the native (blue) and ECMO flow (red) are visualized for four dual drainage cases. The data were averaged over the last second of simulation. *Abbreviations*: FF, femoro-femoral; JF, jugulo-femoral; FJ, femoro-jugular. In dual drainage (JFF): **ss**, single stage jugular cannula; **ms**, multistage; subscripts 1, 2 and 3 indicate the jugular drainage flow rate (L/min); subscripts f + , j- and f-f- indicate the advancement (+) or retraction (-) of the jugular (j) or femoral (f) cannulae.

Recirculation fractions for each studied configuration, distinguishing between the jugular and femoral cannula contributions in dual drainage (JFF) cases, are shown in Fig 3.

Draining 1 L/min from the single stage jugular cannula in dual drainage mode (JFF**ss**${\;}_{1}$) exhibited a higher R${\;}_{f}$ (21.1%) compared to single drainage configurations FF (10.6%) and FJ (17.5%), with the highest contribution to R${\;}_{f}$ coming from the jugular cannula. When draining 2L/min (JFF**ss**${\;}_{2}$) all the recirculation occurred via the jugular cannula, with R${\;}_{f}$ (17.1%) similar to FJ but still higher than seen for FF. Retracting the single stage cannula in the SVC to a position 2 cm above the superior cavo-atrial junction reduced R${\;}_{f}$ by 16% in both the above mentioned cases. The effect of this repositioning is visualized with streamlines of ECMO and native venous blood flow for JFF**ss**${\;}_{1}$ in Fig 3B.

Using a multistage cannula in the SVC (Fig 3C), showed a R${\;}_{f}$ of 0.03% at a cannula drainage flow rate of 2 L/min. Increasing the drainage flow to 3 L/min, which was above the incoming SVC flow (2.7 L/min), led to an increase in R${\;}_{f}$ to 15%. The streamlines of the ECMO and native flow for these two cases are compared in Fig 3C. Retracting the two cannulae placed in the IVC, return and drainage, had no significant impact on R${\;}_{f}$, Fig 3C.

**Fig 4:**
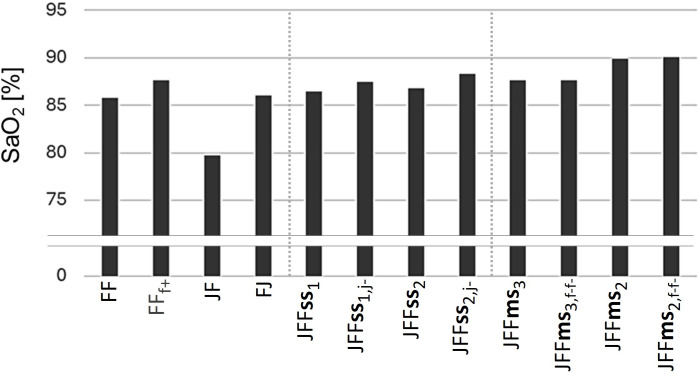
Arterial oxygen saturation, SaO${\;}_{2}$. The data were averaged over the last second of simulation. *Abbreviations*: FF, femoro-femoral; JF, jugulo-femoral; FJ, femoro-jugular. In dual drainage (JFF): **ss**, single stage jugular cannula; **ms**, multistage; subscripts 1, 2 and 3 indicate the jugular drainage flow rate (L/min); subscripts f + , j- and f-f- indicate advancement (+) or retraction (-) of the cannula.

The arterial oxygen saturation was highest for JFF**ms** with equal drainage between the jugular cannula and the femoral one (89.9%). Furthermore, in all but one case SaO${\;}_{2}$ was above 85%. The lowest SaO${\;}_{2}$ was noted for JF (79.7%). In FF, pushing the return cannula closer to the RA/SVC junction resulted in a relative increase in SaO${\;}_{2}$ of 2%. In dual drainage, retracting the jugular single stage cannula led to relative increases in SaO${\;}_{2}$ of 1.1% and 1.7%, for unequal and equal drainage respectively. The values of SaO${\;}_{2}$ for each configuration are reported in Fig 4.

### Drainage fractions in the cannula placed in the superior vena cava

**Fig 5:**
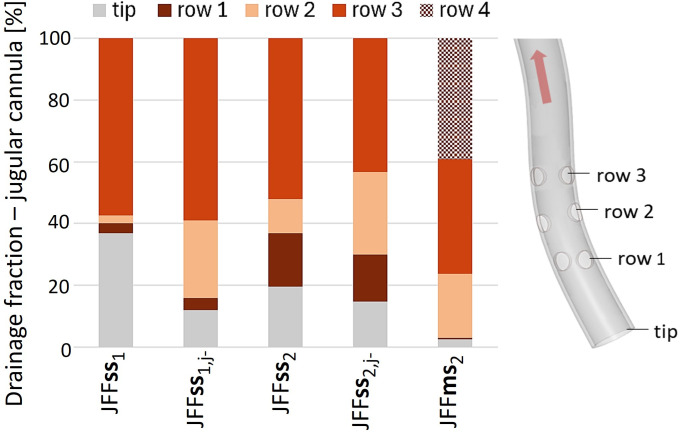
Drainage fractions. Percentage of blood flow drained from the tip and from the side hole-rows of the jugular cannula (row 1, 2 and 3 are depicted on the right for the single stage cannula, while row 4 refers to the most proximal hole-row of the multistage cannula). *Abbreviations*: In dual drainage (JFF): **ss**, single stage jugular cannula; **ms**, multistage; subscripts 1 and 2 indicate the jugular drainage flow rate (L/min); subscript j- indicates retraction of jugular cannula.

In all dual drainage cases with single stage jugular cannula (JFF**ss**), the highest contribution to drainage came from the most proximal row of holes (row 3, the closest to the pump), as shown in Fig 5. When draining 1 L/min (JFF**ss**${\;}_{1}$), the jugular cannula drained 37.0% from its tip, which decreased to 12.0% when retracting the cannula.

Draining 2 L/min with a multistage jugular cannula allowed to reduce the drainage fraction at its tip and most distal hole-row, compared to a single stage type, as depicted in Fig 5 (JFF**ms**${\;}_{2}$ vs. JFF**ss**${\;}_{2}$).

### Caval pressures

**Fig 6:**
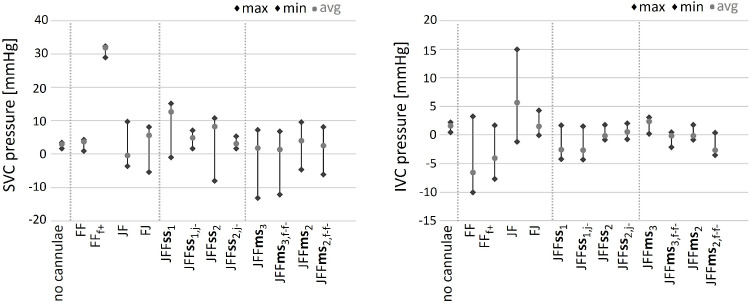
Caval pressures. Minimum, average and maximum values of pressures at the superior vena cava (SVC) and inferior vena cava (IVC) walls, averaged over the last second of simulation. *Abbreviations*: FF, femoro-femoral; JF, jugulo-femoral; FJ, femoro-jugular. In dual drainage (JFF): **ss**, single stage jugular cannula; **ms** multistage, subscripts 1, 2 and 3 indicate the jugular drainage flow rate (L/min); subscripts f + , j- and f-f- indicate advancement (+) or retraction (-) of the cannula.

The minimum, average and maximum pressures at the wall of SVC and IVC are reported in Fig 6. The baseline values, with no cannulae inserted, ranged from 1.6 to 3.5 mmHg at the SVC, and from 0.4 to 2.2 mmHg at the IVC.

#### Superior vena cava pressure.

The surface-averaged values of SVC pressures were positive in all the studied configurations except for JF (−0.4 mmHg). In FF configuration, the average SVC pressure increased from 3.8 to 32 mmHg when the return cannula was pushed up to the superior cavo-atrial junction (FF${\;}_{f+}$, S1 File). The minimum SVC pressures were negative in JF, FJ and in all JFF cases, except when the jugular single stage cannula was repositioned.

In dual drainage mode, retracting the jugular single stage cannula caused a decrease in average pressure (from 12.6 to 4.9 mmHg in JFF**ss**${\;}_{1}$ and from 8.3 to 3.2 mmHg in JFF**ss**${\;}_{2}$), along with an increase in minimum pressure. When using a multistage jugular cannula in dual drainage configurations, the minimum pressures were localized in the vicinity of the most proximal side holes, while the maximum pressures were captured at the upper part of the right brachial vein.

#### Inferior vena cava pressure.

The lowest IVC pressure was noted for FF configuration with −10 mmHg at the level of the most proximal row of holes. The highest maximum IVC pressure, 15 mmHg, was obtained for JF (return zone).

In dual drainage configurations, retracting the return and femoral drainage cannulae by 3.5 cm caused a decrease in the IVC average pressure of approximately 2.5 mmHg.

### Shear rates and wall shear stresses

**Fig 7:**
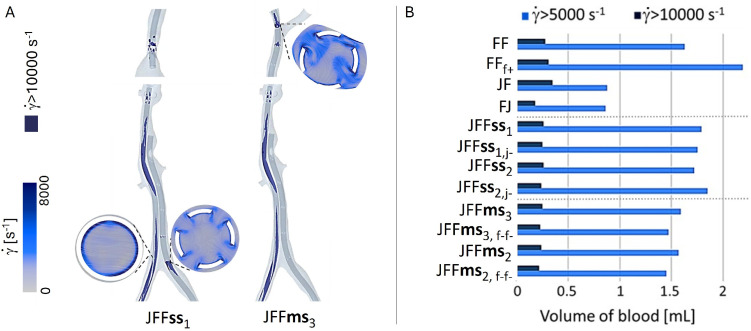
Shear rates, $\dot{\gamma }$. A) Visualization of locations with $\dot{\gamma }>$10000 s^-1^, along with details of $\dot{\gamma }$ on cross sectional planes of the return and drainage cannula in the inferior (JFF**ss**${\;}_{1}$) and superior vena cava (JFF**ms**${\;}_{3}$). B) Volume of blood with $\dot{\gamma }$ exceeding 5000 and 10000 s^-1^ for the studied configurations. Data were averaged over the last second of simulation. *Abbreviations*: FF, femoro-femoral; JF, jugulo-femoral; FJ, femoro-jugular; JFF, dual drainage: **ss**, single stage jugular cannula; **ms**, multistage; subscripts 1, 2 and 3 indicate the jugular drainage flow rate (L/min); subscripts f + , j- and f-f- indicate the advancement (+) or retraction (-) of the cannula.

For all the studied configurations, the highest values of $\dot{\gamma }$, > 10000 s^-1^, were localized along the inner wall of the return cannulae and at the most proximal row of drainage holes. For dual drainage configurations, the latter were predominant either in the jugular or femoral cannula, depending on the drainage flow ratio. To exemplify, two cases are shown in Fig 7A. The average volume of blood with $\dot{\gamma }>$10000 s^-1^ was equal to 0.24 mL for JFF configurations.

The volume of blood characterized by shear rate $\dot{\gamma }>$5000 s^-1^ was equal to 1.78 mL (1.16% of the total blood volume of the patient-averaged geometry) on average for the JFF**ss** cases and 1.52 mL (1%) for the JFF**ms** configurations. The FF configuration with the return cannula pushed until the SVC-RA junction (FF${\;}_{f+}$) presented the highest value (2.19 mL, 1.43%), while FJ the lowest (0.86 mL, 0.56%). The values for each configuration are reported in Fig 7B.

For baseline condition, the highest values of $\dot{\gamma }$, 3500−4000 s^-1^, were localized at the superior cavo-atrial junction.

**Fig 8:**
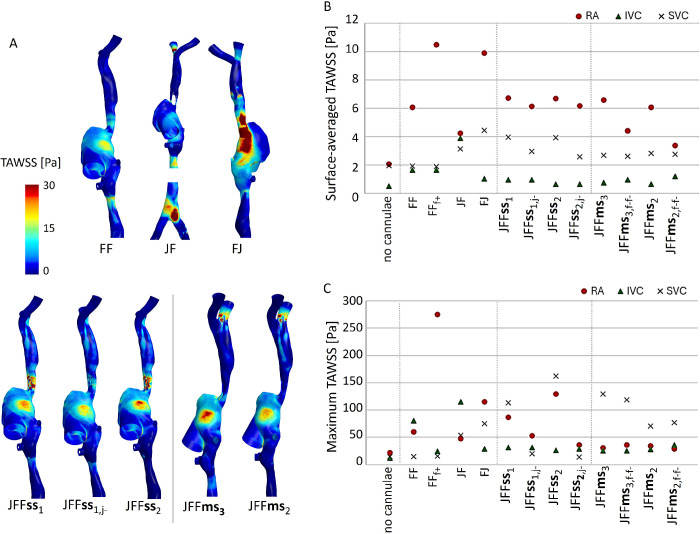
Time averaged wall shear stress, TAWSS. A) Colour contour plot for single drainage configurations (FF, JF, FJ), JFF**ss** (JFF**ss**${\;}_{1}$, JFF**ss**${\;}_{1,j-}$, JFF**ss**${\;}_{2}$) and JFF**ms** (JFF**ms**${\;}_{3}$, JFF**ms**${\;}_{2}$). Cut views. B) Surface-averaged TAWSS on the right atrium (RA), inferior vena cava (IVC) and superior vena cava (SVC). C) Maximum TAWSS on RA, IVC and SVC. *Abbreviations*: FF, femoro-femoral; JF, jugulo-femoral; FJ, femoro-jugular; JFF, dual drainage: **ss**, single stage jugular cannula; **ms**, multistage; subscripts 1, 2 and 3 indicate the jugular drainage flow rate (L/min); subscripts f + , j- and f-f- indicate the advancement (+) or retraction (-) of the cannula.

The surface-averaged values of TAWSS on the RA, IVC and SVC for the studied configurations are depicted in Fig 8B. In the baseline case, the surface-averaged TAWSS were 2 Pa on the RA wall, 0.5 Pa on the IVC and 2 Pa on the SVC. For all the studied cannulation configurations, the highest surface-averaged TAWSS were found in the RA, with peak values for FF${\;}_{f+}$ and FJ (10 Pa).

**Fig 9:**
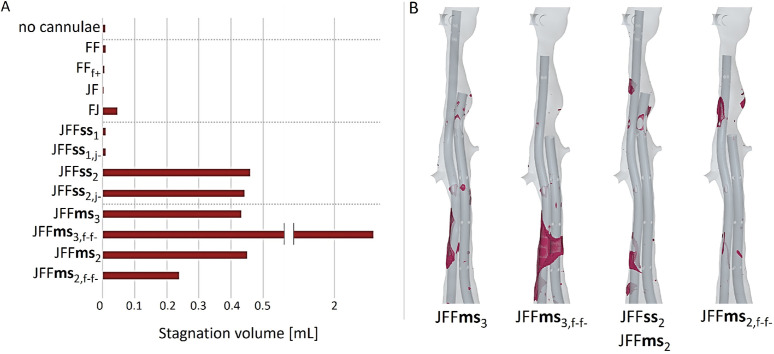
Stagnation volume. A) Blood volume of stagnation (average blood flow velocity *u* < 1 mm/s and shear rate $\dot{\gamma }<$2*s*^-1^). B) Stagnation zones for five dual drainage cases. Data were averaged over the last second of simulation. *Abbreviations*: FF, femoro-femoral; JF, jugulo-femoral; FJ, femoro-jugular; JFF, dual drainage: **ss**, single stage jugular cannula; **ms**, multistage; subscripts 1, 2 and 3 indicate the jugular drainage flow rate (L/min); subscripts f + , j- and f-f- indicate the advancement (+) or retraction (-) of the cannula.

The location and values of maximum TAWSS are depicted in Fig 9A and 9C. In FF, the max TAWSS (81 Pa) was located on the IVC at the level of the most proximal hole-row of the return cannula (Fig 8A, 9C). In FF${\;}_{f+}$, TAWSS reached a maximum of 275 Pa, at the superior cavo-atrial junction, where the flow jet from the return cannula impinged on the RA wall (S1 File). For JF the peak values were at the iliac bifurcation, where the return flow jet impacted the wall (59 Pa) and close to the holes of the return cannula (115 Pa). The areas with the highest TAWSS were located where the jet from the return cannula impinged on the wall also for case FJ, at the superior and inferior cavo-atrial junctions, with max value (115 Pa) near the tricuspid valve. In JFF**ss**, the highest values were found at the level of the drainage holes of the jugular cannula (max 113 Pa for JFF**ss**${\;}_{1}$ and 162 Pa for JFF**ss**${\;}_{2}$). These values decreased to 20 Pa and 14 Pa, respectively, when the jugular cannula was retracted 2 cm. In JFF**ms**, the highest TAWSS was located at the level of most proximal hole-row of the jugular drainage cannula.

### Stagnation volume

The highest stagnation volume (2.38 mL) occured in dual drainage mode when draining 1 L/min from the femoral cannula in retracted position (JFF**ms**${\;}_{3,f-f-}$), as depicted in Fig 9. The larger stagnation regions were localized in the IVC, especially in narrow spaces between the return cannula and the vein wall, Fig 9B.

The location and amount of stagnation was independent of the type of jugular cannula, at the same drainage ratio (JFF**ss**${\;}_{2}$ vs. JFF**ms**${\;}_{2}$). Retracting the femoral cannulae led to an increase in the stagnation volume when draining 1 L/min at IVC (JFF**ms**${\;}_{3,f-f-}$), while a decrease occurred when draining 2 L/min (JFF**ms**${\;}_{2,f-f-}$).

## Discussion

In this work, conventional single drainage (single-lumen cannula) VV ECMO configurations were compared with different dual drainage configurations in a patient-averaged digital 3D geometry of the central venous system. From CFD simulations, data on oxygenation efficiency, vascular wall pressures, shear strains, TAWSS and stagnation areas were retrieved and compared.

### Oxygenation: Effect of drainage ratio, cannulae type and position

Among the single drainage configurations, FF with the repositioned return cannula showed the lowest amount of R${\;}_{f}$ and the highest SaO${\;}_{2}$.

R${\;}_{f}$ in a dual drainage configuration was influenced by the type of cannula used in the SVC (single stage vs. multistage), its position, the drainage ratio between the jugular and femoral cannulae, and the available venous return vs. the blood flow needed to be drained in the respective vena cava. Where the drainage flow rate exceeded the venous inflow, higher values of recirculation fraction occured. This phenomenon was observed in previous works on single drainage cannulation [17] and in the present study with dual drainage cannulation where R${\;}_{f}$ increased from virtually nil to 15% when increasing the jugular drainage flow from 2 to 3 L/min in a case where the availability of incoming native venous return was 2.7 L/min in the SVC (JFF**ms**${\;}_{2}$ vs. JFF**ms**${\;}_{3}$ in Fig 3C). In a clinical situation, for an efficient ECMO support, it is advisable to choose a drainage site with high venous inflow from the periphery. In case of similar native inflow, drainage should be aimed to capture the least saturated blood to maximize the oxygen transfer rate over the membrane lung.

In our investigation on dual drainage, when the drainage flow rate was lower than the incoming venous flow, the drainage cannula design became important. Choosing a multistage jugular cannula instead of a single stage reduced R${\;}_{f}$ and increased SaO${\;}_{2}$. The single stage cannula presented a higher drainage fraction at the tip and most distal row of side holes compared to the multistage (Fig 5), which contributed to increase the drainage of mixed blood (native and ECMO) in the RA, thus lowering the oxygenation performance of the treatment.

Regarding the effect of cannula positioning, the single stage jugular cannula performed better, both in terms of R${\;}_{f}$ and SaO${\;}_{2}$, when the cannula to wall distance was larger (JFF**ss**${\;}_{j-}$ vs. JFF**ss**, Figs 3B and 4). Indeed, the effect of moving the jugular cannula 2 cm up from the superior cavo-atrial junction in this geometry translated in restoring a gradually increasing drainage fraction from the distal to the most proximal hole-row, while reducing the drainage fraction at the tip, thus, increasing the drainage of native venous blood (Fig 5). On the other side, increasing the distance between the return cannula in the IVC and the multistage drainage cannula in the SVC had less effect on R${\;}_{f}$ and SaO${\;}_{2}$ (JFF**ms**${\;}_{f-f-}$ vs. JFF**ms**, Figs 3C and 4).

In summary, when comparing single and dual drainage configurations, SaO${\;}_{2}$ was consistently lower in single drainage cases (except for FF${\;}_{f+}$), although adding a drainage cannula did not necessarily lower R${\;}_{f}$ when compared to FF and FJ scenarios.

### Hemodynamic parameters: Effect of drainage ratio, cannulae type and position

Negative pressures in the IVC were more prone to develop in FF and JFF with dominant drainage fraction from the femoral cannula (JFF**ss**${\;}_{1}$). This may result in drainage problems where the cannula may cling to the vascular wall causing drainage tubing chattering. When the drainage cannula is placed in a narrow and/or low-compliant anatomical volume (neonates, small children, intrathoracic processes affecting RA volume, etc.), this is a common problem, which also could develop in FJ and JF [15,35]. The issue is usually resolved with volume administration to the patient, which correlates with negative outcomes [36]. Moreover, fault diagnosis is necessary and cannula adjustment, or another intervention may be needed [37]. According to previous clinical studies, JF is less sensitive to hypovolemia than FJ configuration, and in the latter, multistage cannulae resulted in improved fluid balance as compared to single stage cannulae [15,38,39]. Obstruction of the IVCs by large ECMO cannulae or high ECMO return flows (along with low or no drainage flow at that site, e.g., in JF) with subsequent risk of venous pressure increase in the IVC, and in renal veins, may impact development of acute kidney injury and thus morbidity and mortality [28,29]. Similarly, aiming for a high position of a femoral return cannula, illustrated by FF${\;}_{f+}$ in this study, where the SVC pressure ranged between 29 and 32 mmHg, may be dangerous. Obstruction of the SVC may clinically be part of a not well-recognized problem. Using a 31 Fr bicaval dual-lumen cannula instead of a 27 Fr carried a 2.7 times higher odds ratio for intracranial bleeding [40], and SVC syndrome on VV ECMO has also been reported [41].

Regarding shear rates and stresses, while literature agrees that high values cause blood damage (degradation of von Willebrand factor (vWf), platelet activation, and hemolysis), different critical values have been reported. While the threshold depends on the considered blood component (vWf, platelets, red blood cells), Chan et al. found that to completely avoid any form of blood trauma, shear stress should be below 12 Pa [42]. According to Casa et al., shear rates >5000 s^-1^ are generally considered pathologic [43]. Moreover, the type of flow may influence the above-mentioned threshold [33]. Shear rates analysis showed regions with $\dot{\gamma }>$10000 s^-1^ in all configurations. These were localized at the inner walls of the return cannulae, and at the most proximal row of holes of the drainage cannulae, Fig 7. This suggests that the current cannulae may benefit from holes re-design to reduce blood trauma, also highlighted in the work by Vatani et al. [23].

On the other hand, regions characterized by flow stasis and low shear rates are also known to have thrombotic potential [44]. In the present study, the stagnation volume was mainly dependent on the drainage ratio (similar values and location were obtained for JFF**ss**${\;}_{2}$ and JFF**ms**${\;}_{2}$). When reducing the femoral drainage flow from 2 to 1 L/min the stagnation volume increased, and hence the risk of thrombus formation and subsequent embolization. Emboli may break free during ongoing support or explantation of ECMO, when the cannulae are pulled out [4,45]. In our study, the effect of retracting the return and drainage cannulae in the IVC was linked to the drained flow rate. At a femoral drainage flow of 2 L/min, retraction of both cannulae lowered the stagnating volume, while the opposite occurred when drainage was reduced (Fig 9). Although the maximum stagnation volume was 1.5% of the total blood volume, worst case, it should be considered that this value may increase for different cardiac outputs and ECMO flow rates. Moreover, although 0.5 mL may seem like a small blood volume, around 1-2x10^8^ platelets can potentially accumulate in this volume. In flow stagnation-zones, the choice of anticoagulation management may have great impact on patient risk and thrombus formation. Bivalirudin, a direct thrombin inhibitor, is metabolized by proteolytic plasma enzymes and in stagnated areas bivalirudin is consumed and anticoagulation lost [46].

All the tested configurations exposed the wall of RA and central veins to average TAWSS around two times higher than baseline, Fig 8. JF, and JFF with dominating jugular drainage and retracted femoral cannulae seemed to be the most gentle approaches. The maximum TAWSS (275 Pa) was seen in the RA when the return cannula was pushed until the superior cavo atrial junction. These supra-physiological stresses may lead to damage or morphological alteration of endothelial cells and platelet activation [47,48].

The FF${\;}_{f+}$ case illustrated how a suboptimal position of the return cannula affect the overall dynamics: while pushing the return cannula up to the superior cavo-atrial junction allowed to slightly decrease R${\;}_{f}$ and increase SaO${\;}_{2}$, the jet from the return cannula impinged against the narrow superior cavo-atrial junction leading to a significant increase in SVC venous pressure, shear strains and TAWSS, well above physiological/baseline values (S1 File).

Although not exhaustive, this study contributes to complement the knowledge about dual drainage cannulation present in literature, currently limited to few clinical reports showing improved oxygenation in cases of refractory hypoxemia [10,11]. Moreover, the results explain how different cannulation strategies and choice of cannulae impact the outcome in terms of R${\;}_{f}$ and hemodynamics, important knowledge in improving the treatment.

### Limitations

This work presents some modeling simplifications: the veins and RA were considered rigid, and the boundary conditions were assumed constant. Neither the breathing cycles (spontaneous breathing with ambient pressures or mechanical ventilation with positive pressures), nor the cardiac pulsatility were modeled, hence their impact on oxygenation performance and hemodynamic parameters were not considered. The sensitivity of the model to transient boundary conditions at the venous inlets and at the tricuspid valve could be addressed in future works. However, it is worth noting that the pulsatility in the venous vascular compartment is lower than in the arterial side. A previous work on RA hemodynamics (with no ECMO applied) showed a limited impact of transient boundary conditions on time-averaged velocity in the RA [49]. Conrad and Wang [50] proposed a fluid-structure interaction model for FJ configuration, however the results were not compared against rigid-wall CFD. In addition, they observed that the change in atrial volume had a minimal influence on the recirculation fraction (<5%).

The estimation of arterial oxygen saturation is based on a simplified model. The venous oxygen saturation is subject to clinical variability [51]. Furthermore, the variation in hemoglobin affinity for oxygen, influenced by factors such as partial pressure of oxygen, temperature, and pH (as described by the nonlinear oxygen dissociation curve) has not been considered in the current model [52]. Therefore, the reported SaO${\;}_{2}$ values should be interpreted comparatively rather than as absolute clinical predictions.

In the present study, a supine position was assumed based on clinical preference. Thus, no adjustment was performed regarding hydrostatic pressure influence due to gravity. Moreover, the analyses were conducted in a patient-averaged model with a set ECMO flow rate of 4 L/min and a cardiac output of 6 L/min, thus limiting the span of possible clinical scenarios. The distribution of cardiac output between the SVC and IVC, not clinically available, was retrieved from literature [53,54]. Despite assumptions on boundary conditions, prior comparison against clinical data indicated that the current model well captured average values of recirculation fraction for single drainage configurations (JF and FJ) [55]. A smooth ECMO run, characterized by a constant ECMO flow rate and ideal conditions, was herein simulated. However, in the clinic, problems related to drainage can occur and/or an increase in ECMO flow rate can be considered to meet oxygenation targets.

Patient-specific anatomical variations may quantitatively alter the results presented, though it is reasonable to assume that the trends and discussed comparisons hold. The positions of the cannulae were imposed to follow the centerline of the veins, which is a simplification of reality. The side holes of the cannulae were assumed to be fully patent, except when the cannula cross-section exceeded that of the veins.

Future directions include assessing the impact of RA motion and vessel wall compliance on the variables of interest and investigating the effect of changes in cannula design on hemodynamic and oxygenation performance. Exposure and residence times are also important factors for blood damage and flow-induced thrombosis, not the least considering flow stagnation zones and anticoagulation management, and they could be estimated in future developments.

## Conclusion

In this work, dual drainage cannulation was computationally investigated and compared against single drainage configurations (for a given ECMO flow rate and cardiac output). The results suggested that the type of the additional jugular drainage cannula (single stage vs. multistage), its position and the drainage ratio between the jugular and femoral cannula affect R${\;}_{f}$. For some dual drainage configurations R${\;}_{f}$ may become higher than in femoro-femoral and femoro-jugular single drainage configurations. An additional single stage drainage cannula placed in the SVC allowed to lower the recirculation faction with respect to conventional single drainage cases only if properly positioned. In dual drainage mode, adding a multistage cannula in the SVC led to the best oxygenation performance, among all studied configurations, when draining at a flow rate lower than the native venous inflow.

However, while it is important to optimize oxygenation, the flow characteristics and related hemodynamic parameters (caval pressures, stress dynamics including shear rates and TAWSS, stagnation areas) are also important for improved VV ECMO treatment and patient outcome.

## Supporting information

S1 File Details of the geometry. Computational fluid dynamics setup. Grid convergence study. Effect of pushing the return cannula until the superior cavo-atrial junction in femoro-femoral configuration. (PDF)

## References

[1] Abrams D, Bacchetta M, Brodie D. Recirculation in venovenous extracorporeal membrane oxygenation. ASAIO J. 2015;61(2):115–21. doi: 10.1097/MAT.0000000000000179 25423117

[2] Gagliardi V, Gagliardi G. Recirculation in Veno-Venous Extracorporeal Membrane Oxygenation. Medicina (Kaunas). 2024;60(12):1936. doi: 10.3390/medicina60121936 39768819 PMC11676060

[3] Extracorporeal Life Support Organization Guidelines for Cardiopulmonary Extracorporeal Life Support. Ann Arbor, MI, USA. 2017. http://www.elso.org

[4] Abruzzo A, Gorantla V, Thomas SE. Venous thromboembolic events in the setting of extracorporeal membrane oxygenation support in adults: A systematic review. Thromb Res. 2022;212:58–71. doi: 10.1016/j.thromres.2022.02.015 35219933

[5] Nunez JI, Gosling AF, O’Gara B, Kennedy KF, Rycus P, Abrams D, et al. Bleeding and thrombotic events in adults supported with venovenous extracorporeal membrane oxygenation: an ELSO registry analysis. Intensive Care Med. 2022;48(2):213–24. doi: 10.1007/s00134-021-06593-x 34921625 PMC9178906

[6] Teijeiro-Paradis R, Gannon WD, Fan E. Complications associated with venovenous extracorporeal membrane oxygenation—what can go wrong?. Critical Care Medicine. 2022;50(12):1809–18. 36094523 10.1097/CCM.0000000000005673

[7] Ostermann M, Lumlertgul N. Acute Kidney Injury in ECMO Patients. Annual Update in Intensive Care and Emergency Medicine. Springer International Publishing. 2021. 207–22. 10.1007/978-3-030-73231-8_18

[8] Villa G, Katz N, Ronco C. Extracorporeal Membrane Oxygenation and the Kidney. Cardiorenal Med. 2015;6(1):50–60. doi: 10.1159/000439444 27194996 PMC4698639

[9] Banfi C, Pozzi M, Siegenthaler N, Brunner M-E, Tassaux D, Obadia J-F, et al. Veno-venous extracorporeal membrane oxygenation: cannulation techniques. J Thorac Dis. 2016;8(12):3762–73. doi: 10.21037/jtd.2016.12.88 28149575 PMC5227239

[10] Ichiba S, Peek GJ, Sosnowski AW, Brennan KJ, Firmin RK. Modifying a venovenous extracorporeal membrane oxygenation circuit to reduce recirculation. Ann Thorac Surg. 2000;69(1):298–9. doi: 10.1016/s0003-4975(99)01227-8 10654547

[11] Hori D, Noguchi K, Nomura Y, Tanaka H. Extracorporeal membrane oxygenation with double venous drainage. Ann Thorac Cardiovasc Surg. 2014;20 Suppl:836–8. doi: 10.5761/atcs.cr.12.01993 23445789

[12] Charbit J, Courvalin E, Dagod G, Deras P, Laumon T, Girard M, et al. Mathematical modelling of oxygenation under veno-venous ECMO configuration using either a femoral or a bicaval drainage. Intensive Care Med Exp. 2022;10(1):10. doi: 10.1186/s40635-022-00434-x 35347456 PMC8960524

[13] Lim SY, Ahn S, Hong S-B, Chung CR, Jeon K, Lee S-M, et al. Clinical outcomes according to cannula configurations in patients with acute respiratory distress syndrome under veno-venous extracorporeal membrane oxygenation: a Korean multicenter study. Ann Intensive Care. 2020;10(1):86. doi: 10.1186/s13613-020-00700-9 32572593 PMC7306930

[14] Pooth J-S, Förster JK, Benk C, Diel P, Brixius SJ, Maier S, et al. Impact of Cannulation Strategy and Extracorporeal Blood Flow on Recirculation During Veno-Venous Extracorporeal Membrane Oxygenation. Artif Organs. 2025;49(6):1012–20. doi: 10.1111/aor.14961 39868656 PMC12120813

[15] Ling SK-H. Comparison of atrio-femoral and femoro-atrial venovenous extracorporeal membrane oxygenation in adult. Perfusion. 2022;37(1):14–8. doi: 10.1177/0267659120969020 33126833

[16] Jung YC, Chong Y, Han SJ, Park S-J, Kwak Y, Kim SY, et al. Impact of Multistage Cannula on Recirculation in Various Venovenous Extracorporeal Membrane Oxygenation Cannulation Strategies. ASAIO Journal. 2025;71(8):667–74. doi: 10.1097/mat.0000000000002384 39963977

[17] Parker LP, Marcial AS, Brismar TB, Broman LM, Prahl Wittberg L. Cannulation configuration and recirculation in venovenous extracorporeal membrane oxygenation. Sci Rep. 2022;12(1):16379. doi: 10.1038/s41598-022-20690-x 36180496 PMC9523655

[18] Khamooshi M, Wickramarachchi A, Byrne T, Seman M, Fletcher DF, Burrell A, et al. Blood flow and emboli transport patterns during venoarterial extracorporeal membrane oxygenation: A computational fluid dynamics study. Comput Biol Med. 2024;172:108263. doi: 10.1016/j.compbiomed.2024.108263 38489988

[19] Leoni M, Szasz J, Meier J, Gerardo-Giorda L. Blood flow but not cannula positioning influences the efficacy of Veno-Venous ECMO therapy. Sci Rep. 2022;12(1):20950. doi: 10.1038/s41598-022-23159-z 36470881 PMC9722702

[20] Palmér O, Palmér K, Hultman J, Broman M. Cannula Design and Recirculation During Venovenous Extracorporeal Membrane Oxygenation. ASAIO J. 2016;62(6):737–42. doi: 10.1097/MAT.0000000000000440 27660904 PMC5098462

[21] Song K, Na SJ, Chung CR, Jeon K, Suh GY, Chung S, et al. Optimal Position of a Femorojugular Cannulation for Venovenous Extracorporeal Membrane Oxygenation in Acute Respiratory Distress Syndrome. Ann Thorac Surg. 2023;115(4):1016–22. doi: 10.1016/j.athoracsur.2022.10.023 36967708

[22] Wickramarachchi A, Khamooshi M, Burrell A, Pellegrino VA, Kaye DM, Gregory SD. The effect of drainage cannula tip position on risk of thrombosis during venoarterial extracorporeal membrane oxygenation. Comput Methods Programs Biomed. 2023;231:107407. doi: 10.1016/j.cmpb.2023.107407 36764061

[23] Vatani A, Liao S, Burrell AJC, Carberry J, Azimi M, Steinseifer U, et al. Improved Drainage Cannula Design to Reduce Thrombosis in Veno-Arterial Extracorporeal Membrane Oxygenation. ASAIO J. 2022;68(2):205–13. doi: 10.1097/MAT.0000000000001440 33883503

[24] Chabry Y, Barry M, Nzomvuama A, Herduin D, Guilbart M, Caus T. ECMO related complications: cannula thrombosis, diagnosis and strategy: a clinical case. BJSTR. 2018.

[25] Morimont P, Lambermont B, Gaspard V, Defraigne J-O. Molding thrombus of an ECMO cannula floating in the right atrium. Intensive Care Med. 2015;41(11):1965–6. doi: 10.1007/s00134-015-3779-0 25851390

[26] Walter JM, Kurihara C, Corbridge TC, Bharat A. Chugging in patients on veno-venous extracorporeal membrane oxygenation: An under-recognized driver of intravenous fluid administration in patients with acute respiratory distress syndrome?. Heart Lung. 2018;47(4):398–400. doi: 10.1016/j.hrtlng.2018.03.011 29681395 PMC6026563

[27] Patel B, Arcaro M, Chatterjee S. Bedside troubleshooting during venovenous extracorporeal membrane oxygenation (ECMO). J Thorac Dis. 2019;11(Suppl 14):S1698–707. doi: 10.21037/jtd.2019.04.81 31632747 PMC6783727

[28] Chen X, Wang X, Honore PM, Spapen HD, Liu D. Renal failure in critically ill patients, beware of applying (central venous) pressure on the kidney. Ann Intensive Care. 2018;8(1):91. doi: 10.1186/s13613-018-0439-x 30238174 PMC6146958

[29] Mori T. Renal venous hypertension to the regulation of pressure natriuresis in heart failure. Hypertens Res. 2024;47(4):1081–3. doi: 10.1038/s41440-023-01512-7 38228752

[30] Parker LP, Svensson Marcial A, Brismar TB, Broman LM, Prahl Wittberg L. Impact of altered vena cava flow rates on right atrium flow characteristics. J Appl Physiol (1985). 2022;132(5):1167–78. doi: 10.1152/japplphysiol.00649.2021 35271411 PMC9054263

[31] Parker LP, Fiusco F, Rorro F, Svensson Marcial A, Brismar TB, Broman LM, et al. Venovenous extracorporeal membrane oxygenation drainage cannula performance: From generalized to patient-averaged vessel model. Physics of Fluids. 2024;36(6). doi: 10.1063/5.0212546

[32] Quemada D. Rheology of concentrated disperse systems II. A model for non-newtonian shear viscosity in steady flows. Rheol Acta. 1978;17(6):632–42. doi: 10.1007/bf01522036

[33] Yeo EF, Oliver JM, Korin N, Waters SL. A continuum model for the elongation and orientation of Von Willebrand factor with applications in arterial flow. Biomech Model Mechanobiol. 2024;23(4):1299–317. doi: 10.1007/s10237-024-01840-8 38592600 PMC11341749

[34] Liao S, Simpson B, Neidlin M, Kaufmann TAS, Li Z, Woodruff MA, et al. Numerical prediction of thrombus risk in an anatomically dilated left ventricle: the effect of inflow cannula designs. Biomed Eng Online. 2016;15(Suppl 2):136. doi: 10.1186/s12938-016-0262-2 28155674 PMC5260141

[35] Frenckner B, Broman M, Broomé M. Position of draining venous cannula in extracorporeal membrane oxygenation for respiratory and respiratory/circulatory support in adult patients. Crit Care. 2018;22(1):163. doi: 10.1186/s13054-018-2083-0 29907121 PMC6003129

[36] Schmidt M, Bailey M, Kelly J, Hodgson C, Cooper DJ, Scheinkestel C, et al. Impact of fluid balance on outcome of adult patients treated with extracorporeal membrane oxygenation. Intensive Care Med. 2014;40(9):1256–66. doi: 10.1007/s00134-014-3360-2 24934814 PMC7094895

[37] Zakhary B, Vercaemst L, Mason P, Lorusso R, Brodie D. How I manage drainage insufficiency on extracorporeal membrane oxygenation. Crit Care. 2020;24(1):151. doi: 10.1186/s13054-020-02870-1 32295631 PMC7161276

[38] Thiara S, Willms A, Isac G, Finlayson G, Kanji H, Romano K, et al. Impact of drainage cannula type on patients’ fluid balance in venovenous extracorporeal membrane oxygenation: A historical cohort study. Perfusion. 2025;40(8):1809–16. doi: 10.1177/02676591251331163 40221843 PMC12602714

[39] Fisser C, Palmér O, Sallisalmi M, Paulus M, Foltan M, Philipp A, et al. Recirculation in single lumen cannula venovenous extracorporeal membrane oxygenation: A non-randomized bi-centric trial. Front Med (Lausanne). 2022;9:973240. doi: 10.3389/fmed.2022.973240 36117961 PMC9470851

[40] Mazzeffi M, Kon Z, Menaker J, Johnson DM, Parise O, Gelsomino S, et al. Large Dual-Lumen Extracorporeal Membrane Oxygenation Cannulas Are Associated with More Intracranial Hemorrhage. ASAIO J. 2019;65(7):674–7. doi: 10.1097/MAT.0000000000000917 30398981

[41] Rajavardhan R, Shetty RM, Achaiah NC, Thimmappa M, Reddy AV. A rare case of superior vena cava syndrome in a patient on VV-ECMO. Indian J Thorac Cardiovasc Surg. 2022;38(2):215–7. doi: 10.1007/s12055-021-01293-z 35002107 PMC8723804

[42] Chan CHH, Simmonds MJ, Fraser KH, Igarashi K, Ki KK, Murashige T, et al. Discrete responses of erythrocytes, platelets, and von Willebrand factor to shear. J Biomech. 2022;130:110898. doi: 10.1016/j.jbiomech.2021.110898 34896790

[43] Casa LDC, Deaton DH, Ku DN. Role of high shear rate in thrombosis. J Vasc Surg. 2015;61(4):1068–80. doi: 10.1016/j.jvs.2014.12.050 25704412

[44] Hathcock JJ. Flow effects on coagulation and thrombosis. Arterioscler Thromb Vasc Biol. 2006;26(8):1729–37. doi: 10.1161/01.ATV.0000229658.76797.30 16741150

[45] Gu Y, Bjelic M, Panda K, Usman AA, Magnuson R, Gosev I. Cannula-Associated Deep Vein Thrombosis After Venovenous Extracorporeal Membrane Oxygenation in Patients with and Without Systemic Anticoagulation. J Cardiothorac Vasc Anesth. 2024;38(1):230–6. doi: 10.1053/j.jvca.2023.09.009 37827919 PMC11788865

[46] Gladwell TD. Bivalirudin: a direct thrombin inhibitor. Clin Ther. 2002;24(1):38–58. doi: 10.1016/s0149-2918(02)85004-4 11833835

[47] Raj N, Pan C, Starke AM, Matos ALL, Soehnlein O, Gerke V. Altered shear stress of blood flow causes plasma membrane damage in endothelial cells. Blood Vessel Thromb Hemost. 2024;2(1):100040. doi: 10.1016/j.bvth.2024.100040 40766873 PMC12320452

[48] Dolan JM, Kolega J, Meng H. High wall shear stress and spatial gradients in vascular pathology: a review. Ann Biomed Eng. 2013;41(7):1411–27. doi: 10.1007/s10439-012-0695-0 23229281 PMC3638073

[49] Parker LP, Svensson Marcial A, Brismar TB, Broman LM, Prahl Wittberg L. Computational Fluid Dynamics of the Right Atrium: A Comparison of Modeling Approaches in a Range of Flow Conditions. Journal of Engineering and Science in Medical Diagnostics and Therapy. 2022;5(3). doi: 10.1115/1.4054526

[50] Conrad SA, Wang D. Evaluation of Recirculation During Venovenous Extracorporeal Membrane Oxygenation Using Computational Fluid Dynamics Incorporating Fluid-Structure Interaction. ASAIO J. 2021;67(8):943–53. doi: 10.1097/MAT.0000000000001314 33315664 PMC8318564

[51] van Beest P, Wietasch G, Scheeren T, Spronk P, Kuiper M. Clinical review: use of venous oxygen saturations as a goal - a yet unfinished puzzle. Crit Care. 2011;15(5):232. doi: 10.1186/cc10351 22047813 PMC3334733

[52] Collins J-A, Rudenski A, Gibson J, Howard L, O’Driscoll R. Relating oxygen partial pressure, saturation and content: the haemoglobin-oxygen dissociation curve. Breathe (Sheff). 2015;11(3):194–201. doi: 10.1183/20734735.001415 26632351 PMC4666443

[53] Kuzo RS, Pooley RA, Crook JE, Heckman MG, Gerber TC. Measurement of caval blood flow with MRI during respiratory maneuvers: implications for vascular contrast opacification on pulmonary CT angiographic studies. AJR Am J Roentgenol. 2007;188(3):839–42. doi: 10.2214/AJR.06.5035 17312076

[54] Wexler L, Bergel DH, Gabe IT, Makin GS, Mills CJ. Velocity of blood flow in normal human venae cavae. Circ Res. 1968;23(3):349–59. doi: 10.1161/01.res.23.3.349 5676450

[55] Hörwing H, Parker L, Svensson-Marcial A, Brismar TB, Broman LM, Prahl Wittberg L. Hemodynamics in femoro-femoral venovenous extracorporeal membrane oxygenation using large eddy simulations. Sci Rep. 2025;15(1):35229. doi: 10.1038/s41598-025-22403-6 41068411 PMC12511417

