# Supplementary material for: Efficiency and hemodynamics of dual drainage venovenous ECMO: A computational parametric study
Source: PLoS One. 2026 Aug 4;21(8):e0354915. doi: 10.1371/journal.pone.0354915 (PMC13436760; doi:10.1371/journal.pone.0354915)
Supplement: Supplementary file 1 [file pone.0354915.s001.pdf]

Efficiency and hemodynamics of dual drainage venovenous ECMO: a computational parametric study

Monica Emendi[1], Hanna Hörwing[1], Louis Parker[2], Anders Svensson Marcial[3,4], Torkel Brismar[3,4]
Lars Mikael Broman[5,6], Lisa Prahl Wittberg[1]

[1]*Department of Engineering Mechanics, KTH Royal Institute of Technology, Stockholm, Sweden
[2]School of Engineering, University of Western Australia, Perth, Australia
[3]Department of Radiology, Karolinska University Hospital and Karolinska Institutet, Stockholm, Sweden
[4]Department of Clinical Science, Intervention and Technology, Karolinska Institutet, Stockholm, Sweden
[5]ECMO Centre Karolinska, Pediatric Perioperative Medicine and Intensive Care, Karolinska University Hospital, Stockholm, Sweden
[6]Department of Physiology and Pharmacology, Karolinska Institutet, Stockholm, Sweden

Details of the geometry

The computational domain, consisting in a patient-averaged model of the right atrium, inferior and superior vena cava, has a total volume of 161.8 mL (42.8 mL for the right atrium). The cross-sectional areas of the model's venous inlets and tricuspid valve are reported in the table below.
More details on the patient-specific geometries can be found in Parker et al. (Table I, Supplementary material)[1].

	**Cross sectional area [cm^2]**
Right brachiocephalic vein	1.38
Left brachiocephalic vein	1.26
Hepatic veins (3 inlets)	0.49
Coronary sinus	0.37
Right renal vein	0.46
Left renal vein	0.12
Right femoral vein	0.86
Left femoral vein	0.87
Tricuspid valve	4.97

Table S1: *Cross-sectional area of the tricuspid valve and venous inlets of the model.*

[1]Parker, Louis P., et al. "Impact of altered vena cava flow rates on right atrium flow characteristics." Journal of Applied Physiology 132.5 (2022): 1167-1178.

The depth of insertion of the cannulae for the different configurations, calculated as the length of the centerline (taken from the respective geometrical inlet boundaries), are the following:

**Insertion depth [cm]**		**Return cannula**	**SVC drainage**	**IVC drainage**
Single drainage	FF	39	-	33
	JF	15	12	-
	FJ	7	-	37
	FF$_{f+}$	46	-	33
Dual drainage	JFF**ss**	39	12	33
	JFF**ss**$_{j-}$	39	10	33
	JFF**ms**	39	12	33
	JFF**ms**$_{f\text{-}f\text{-}}$	35.5	12	29.5

Table S2: *Insertion depth of the return and drainage cannulae in each configuration. FF, femoro-femoral; JF, jugulo-femoral; FJ, femoro-jugular; JFF, dual drainage:* ***ss****, single stage jugular drainage cannula;* ***ms****, multistage; subscripts f+, j- and f-f- indicate the advancement (+) or retraction (-) of the jugular (j) or femoral (f) cannula.*

Computational fluid dynamics setup

Large eddy simulation (LES) with wall-adapting local eddy-viscosity (WALE) sub-grid scale model was implemented with a constant time step of 10^{-4} s and 2nd order central-bounded discretization for the convective term. The simulations were initialized with steady Reynolds-averaged Navier–Stokes (RANS) k–ω model.
The walls were assumed to be rigid, constant flow rates were imposed at the venous inlets (as reported in Table 3 of the main manuscript) and zero pressure was assumed at the tricuspid valve. Extensions with a length about 10 times the inlets/outlets diameter were modeled to obtain a fully developed flow.
The fluid domain was discretized with polyhedral cells with prismatic boundary layer refinement. Three mesh sizes (coarse, medium and fine) were considered for the grid convergence study (details in the next section). Each simulation, with the medium mesh size, was run for about 24 hours on 640 cores of Dardel supercomputer (NAISS, Linköping, Sweden).

Grid convergence study

The grid convergence analysis was carried out for the case JFF**ss**$_2$ (dual drainage cannulation where an equal amount of 2 L/min was drained via the jugular-single stage cannula and femoral cannula).

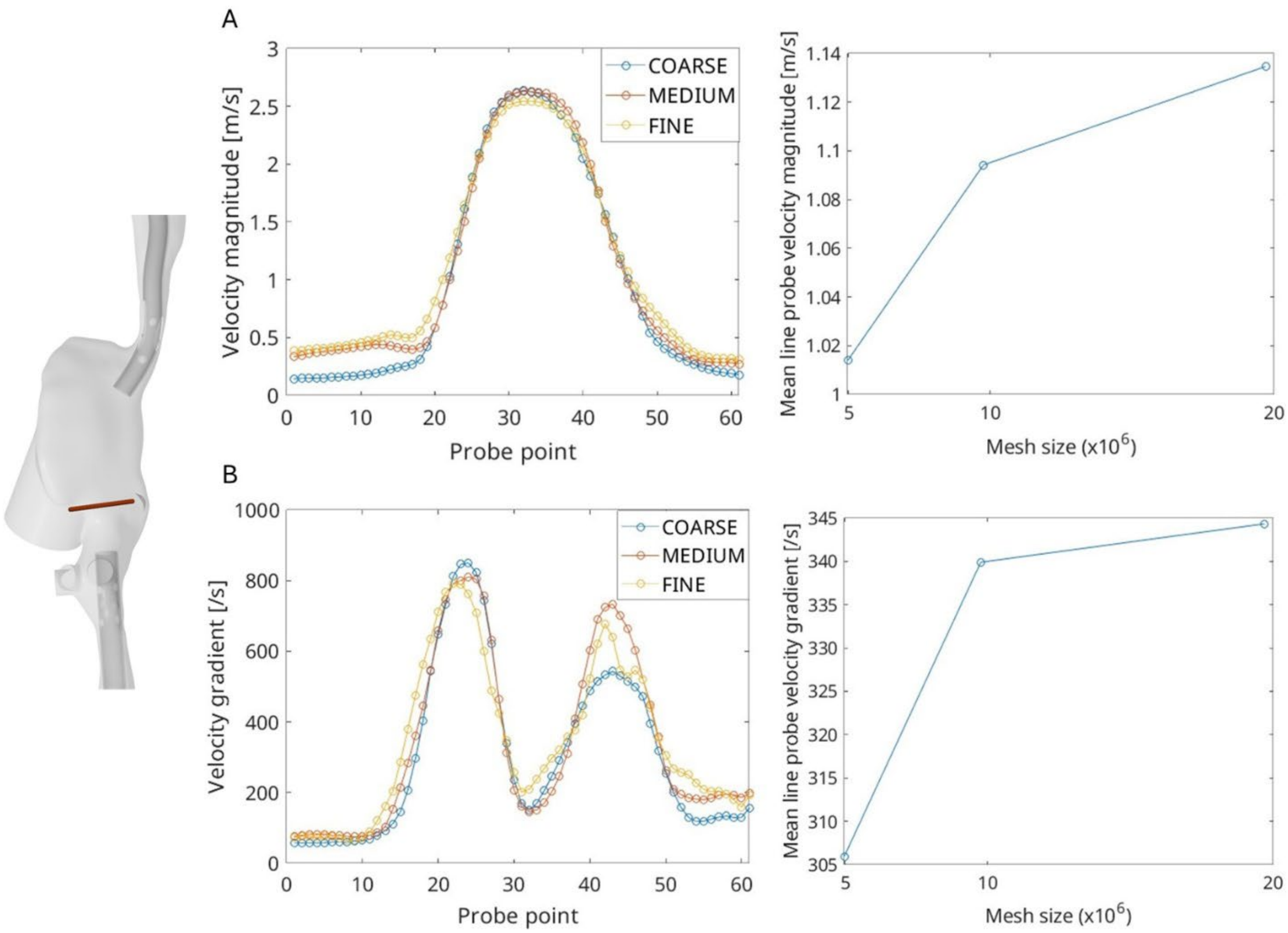

Figure S1: *A) Time-averaged velocity profile along the line probe herein depicted (left) and mean line probe velocity magnitude (right), for the coarse (5M cells), medium (10M cells) and fine (20M cells) meshes. B) Velocity gradient (Frobenius norm) along the line probe and mean line probe velocity gradient (Frobenius norm) for the coarse, medium and fine meshes.*

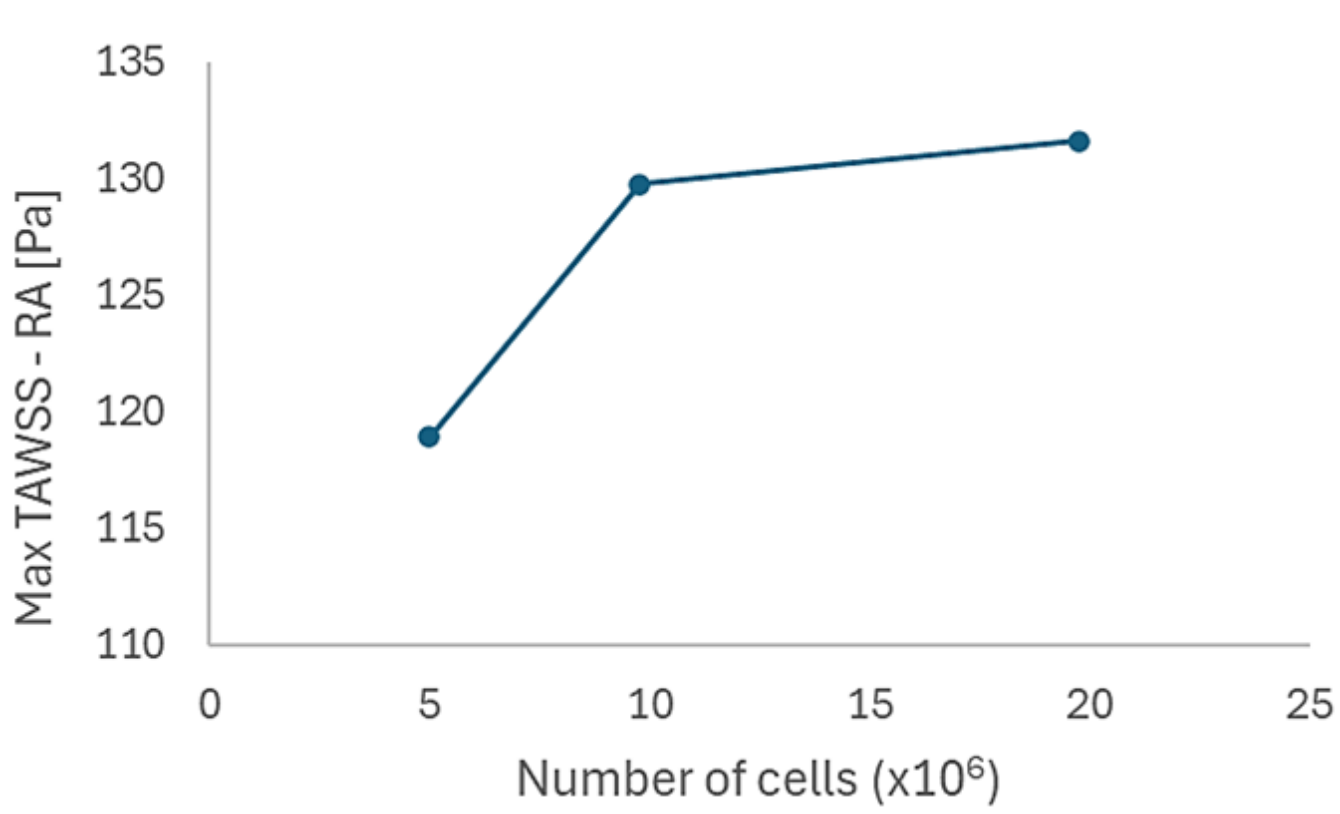

Figure S2: *Maximum values of time-averaged wall shear stress (TAWSS) on the right atrium (RA) for the coarse, medium and fine meshes.*

Grid	Total numbers of cells(x 10^6)	GCI - Max TAWSS (RA)
Fine	19.75	0.35%
Medium	9.76	2.10%
Coarse	4.98	-

Table S3: *Values of grid convergence index (GCI), calculated for the maximum values of time-averaged wall shear stress (TAWSS) on the right atrium (RA), according to Craven et al. (2009)*[2].

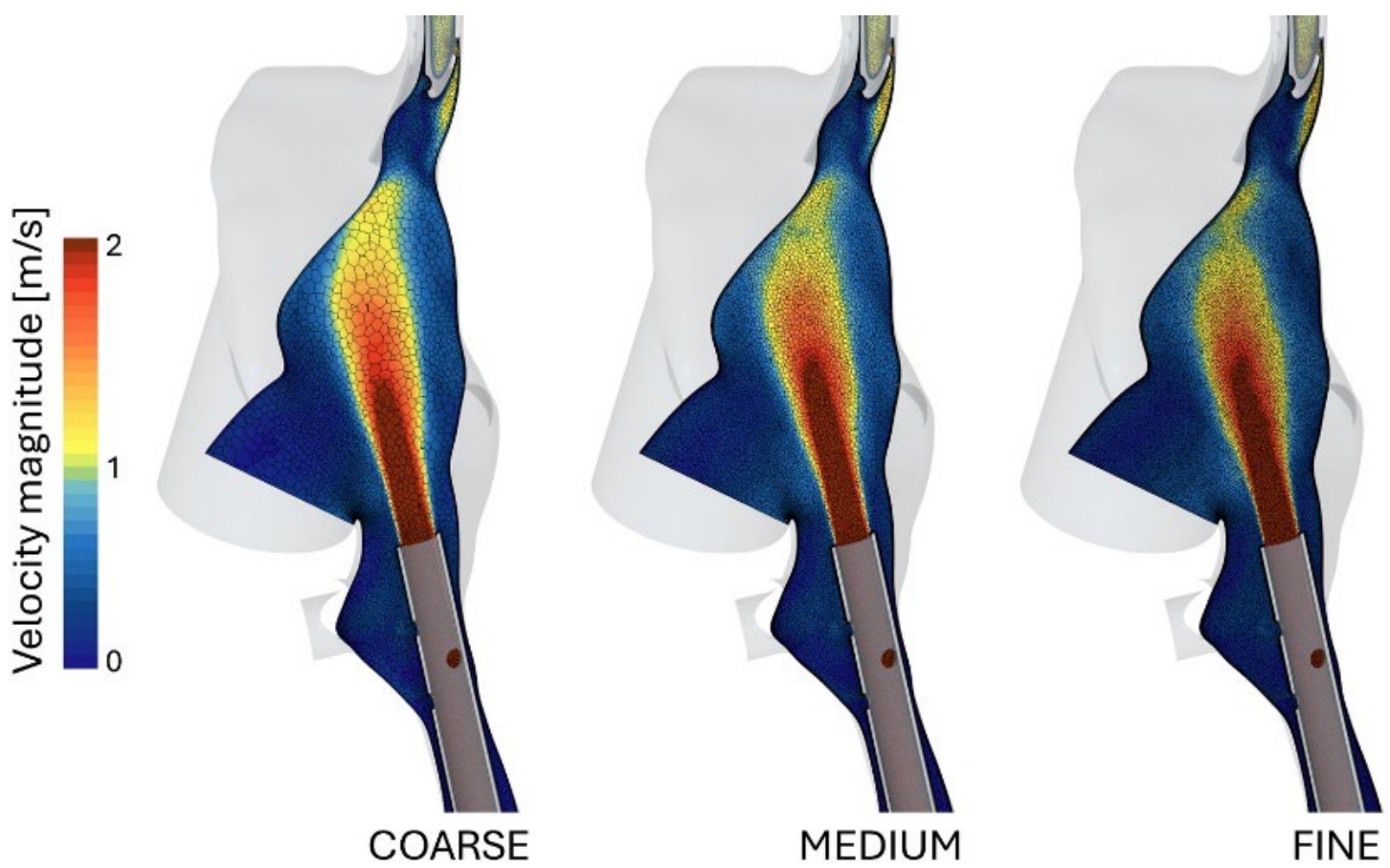

Figure S3: *Velocity magnitude on a cross section through the return cannula and superior vena cava (SVC) drainage cannula, for the coarse, medium and fine meshes.*

[2] Craven BA, Paterson EG, Settles GS, Lawson MJ. Development and verification of a high-fidelity computational fluid dynamics model of canine nasal airflow. J Biomech Eng 131: 091002, 2009.

Effect of pushing the return cannula until the superior cavo-atrial junction in femoro-femoral (FF) configuration

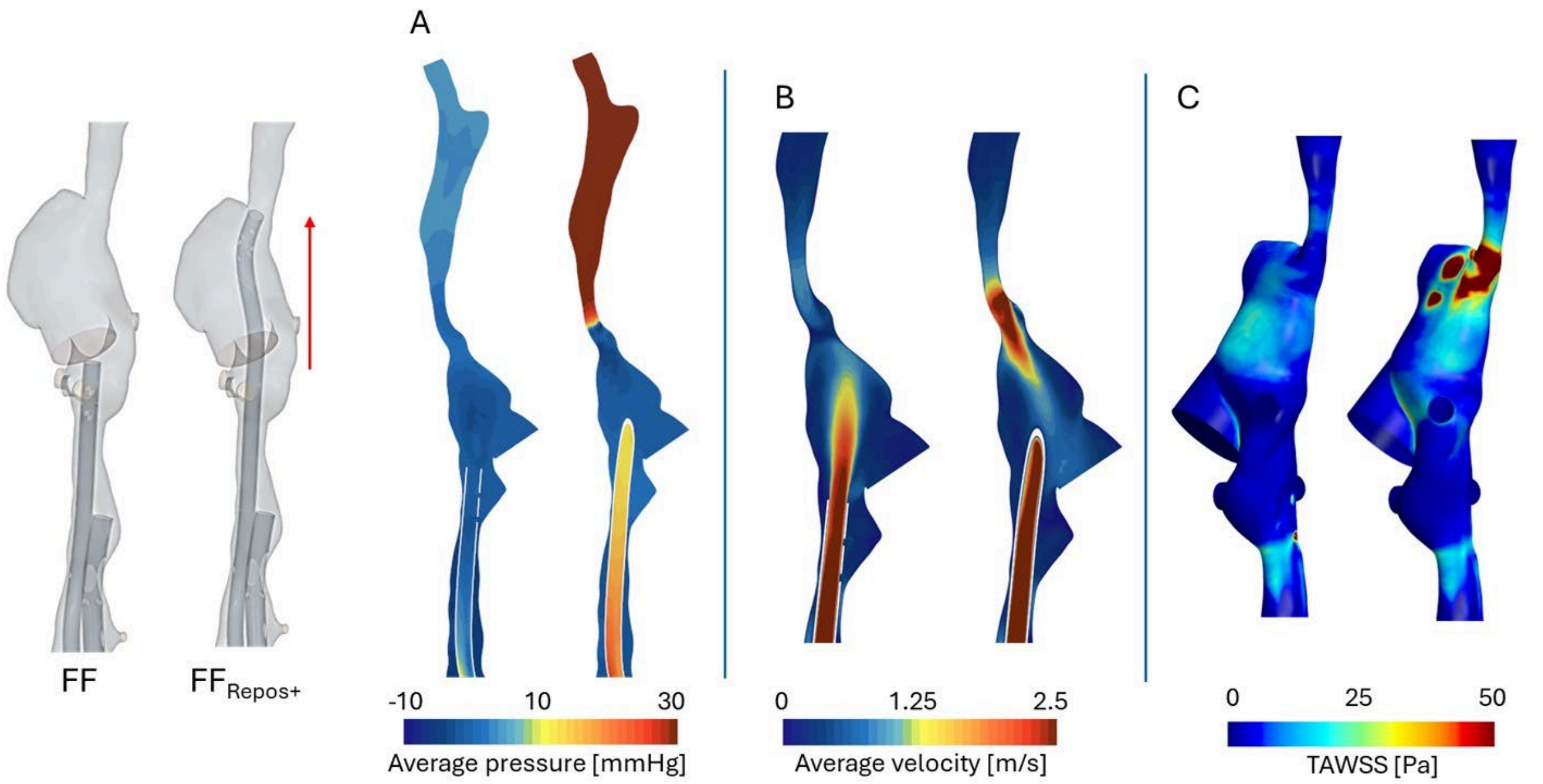

Figure S4: *Comparison femoro-femoral (FF) vs. FF$_{f+}$, where the return cannula was pushed up to the superior cavo-atrial junction as depicted on the left. A) Pressure averaged over the last second of simulation for FF vs. FF$_{f+}$. B) Velocity averaged over the last second of simulation for FF vs. FF$_{f+}$. C) Time averaged wall shear stresses (TAWSS) for FF vs. FF$_{f+}$.*
